# HIV, syphilis, and hepatitis B virus infection and male circumcision in five Sub-Saharan African countries: Findings from the Population-based HIV Impact Assessment surveys, 2015–2019

**DOI:** 10.1371/journal.pgph.0002326

**Published:** 2023-09-18

**Authors:** Megan E. Peck, Megan Bronson, Gaston Djomand, Ikuzo Basile, Kamanzi Collins, Ida Kankindi, Eugenie Kayirangwa, Samuel S. Malamba, Veronicah Mugisha, Sabin Nsanzimana, Eric Remera, Kokuhumbya J. Kazaura, Mbaraka Amuri, Susan Mmbando, George S. Mgomella, Daimon Simbeye, Anna Colletar Awor, Samuel Biraro, Geoffrey Kabuye, Wilford Kirungi, Omega Chituwo, Brave Hanunka, Royd Kamboyi, Lloyd Mulenga, Bupe Musonda, Brian Muyunda, Tepa Nkumbula, Rickie Malaba, John Mandisarisa, Godfrey Musuka, Amy E. Peterson, Carlos Toledo

**Affiliations:** 1 Division of Global HIV & TB, HIV Prevention Branch, Center for Global Health, Centers for Disease Control and Prevention, Atlanta, United States of America; 2 Division of Global HIV & TB, Epidemiology and Surveillance Branch, Center for Global Health, Centers for Disease Control and Prevention, Atlanta, United States of America; 3 Ministry of Health, Rwanda Biomedical Center, Kigali City, Rwanda; 4 ICAP at Columbia University, Kigali City, Rwanda; 5 Division of Global HIV & TB, Center for Global Health, Centers for Disease Control and Prevention, Kigali, Rwanda; 6 Division of Global HIV & TB, Centers for Global Health, Centers for Disease Control and Prevention, Dar-es-Salaam, Tanzania; 7 National AIDS Control Program, Ministry of Health, Dar-es-Salaam, Tanzania; 8 Division of Global HIV & TB, Centers for Global Health, Centers for Disease Control and Prevention, Kampala, Uganda; 9 Columbia University, Kampala, Uganda; 10 Ministry of Health, Kampala, Uganda; 11 Division of Global HIV & TB, Centers for Global Health, Centers for Disease Control and Prevention, Lusaka, Zambia; 12 Department of Public Health, Ministry of Health, Lusaka, Zambia; 13 ICAP at Columbia University, Lusaka, Zambia; 14 Division of Global HIV & TB, Center for Global Health, Centers for Disease Control and Prevention, Harare, Zimbabwe; 15 ICAP at Columbia University, Harare, Zimbabwe; National University of Singapore, SINGAPORE

## Abstract

Voluntary medical male circumcision (VMMC) has primarily been promoted for HIV prevention. Evidence also supports that male circumcision offers protection against other sexually transmitted infections. This analysis assessed the effect of circumcision on syphilis, hepatitis B virus (HBV) infection and HIV. Data from the 2015 to 2019 Population-based HIV Impact Assessments (PHIAs) surveys from Rwanda, Tanzania, Uganda, Zambia, and Zimbabwe were used for the analysis. The PHIA surveys are cross-sectional, nationally representative household surveys that include biomarking testing for HIV, syphilis and HBV infection. This is a secondary data analysis using publicly available PHIA data. Univariate and multivariable logistic regression models were created using pooled PHIA data across the five countries to assess the effect of male circumcision on HIV, active and ever syphilis, and HBV infection among sexually active males aged 15–59 years. Circumcised men had lower odds of syphilis infection, ever or active infection, and HIV, compared to uncircumcised men, after adjusting for covariates (active syphilis infection = 0.67 adjusted odds ratio (aOR), 95% confidence interval (CI), 0.52–0.87, ever having had a syphilis infection = 0.85 aOR, 95% CI, 0.73–0.98, and HIV = 0.53 aOR, 95% CI, 0.47–0.61). No difference between circumcised and uncircumcised men was identified for HBV infection (*P =* 0.75). Circumcised men have a reduced likelihood for syphilis and HIV compared to uncircumcised men. However, we found no statistically significant difference between circumcised and uncircumcised men for HBV infection.

## Introduction

It is well established that medical male circumcision significantly reduces the risk of HIV infection by approximately 60% in males engaging in heterosexual sex [1–3]. In 2007, the World Health Organization (WHO) and the Joint United Nations Program on HIV/AIDS (UNAIDS) first recommended voluntary medical male circumcision (VMMC) as an essential HIV prevention strategy [4,5]. Following this recommendation countries in Eastern and Southern Africa that had generalized HIV epidemics and a low prevalence of male circumcision were prioritized for VMMC. Voluntary medical male circumcision (VMMC) programs offer not only the surgical removal of the foreskin, but also a package of services including screening and referrals for treatment for sexually transmitted infections (STIs) [6,7]. This comprehensive approach is important given that STIs can increase the risk for HIV infection and promote progression of HIV among those living with the disease [8].

Voluntary medical male circumcision (VMMC) programs are promoted primarily for HIV prevention, however, evidence also supports that male circumcision offers protection against other STIs during heterosexual transmission, including syphilis, gonorrhea, and human papillomavirus [9–12]. Epidemiological studies also demonstrate that female partners of medically circumcised men are at a decreased risk for some STIs [13,14]. Further understanding the protective effect of male circumcision on STIs is critical with more than one million STIs acquired globally everyday [15,16].

To date, a multi-country analysis using nationally representative data has not been conducted to estimate the effect of male circumcision on STIs beyond HIV, in countries prioritized for VMMC. To address this gap, this analysis estimated the effect of male circumcision among sexually active men on active and ever syphilis infection, hepatitis B virus (HBV infection), and HIV infection, using data from the Population-based HIV Impact Assessment (PHIA) surveys from 2015 to 2019 conducted in five countries prioritized for VMMC. Additionally, the effect of circumcision by type, medical and non-medical, on STI and HIV infection was assessed.

## Methods

### Study design and population

Population-based HIV Impact Assessments (PHIAs) are cross-sectional, nationally representative household surveys that include biomarking testing for HIV, syphilis and HBV infection. All PHIA surveys employed a 2-stage cluster sampling design to achieve a representative sample of households in each participating country. A core set of standardized questions were included in the PHIAs across all countries that captured information on participant’s demographic, social and behavioral characteristics, household composition, sexual risk-related behaviors, and other HIV related characteristics including asking men to self-report on their circumcision status [17]. Greater details about the PHIA methods, survey design, and testing strategies are described elsewhere [18–22]. Publicly available data from countries prioritized for VMMC that measured HBV or syphilis infection were included in our analysis. Data from Rwanda (2018–2019), Tanzania (2016–2017), Uganda (2016–2017), Zambia (2016) and Zimbabwe (2015–2016) were pooled [23]. Zimbabwe did not measure HBV infection, and Rwanda did not measure syphilis infection and were omitted from the analysis specific to those respective outcomes. Human subjects and ethical approval for each PHIA survey was granted by in-country ethical review committees and institutional review boards at Columbia University Medical Center, Westat, and the U.S. Centers for Disease Control and Prevention. Ethical approval numbers included the following: Rwanda RPHIA-7157, Tanzania, THIS-6880, Uganda, UPHIA-6830, Zambia, ZAMPHIA-6720, Zimbabwe, ZIMPHIA-6702. Formal informed consent was obtained from each participant prior to data collection. The authors of this analysis did not have access to information that could identify individual participants during or after data collection.

### Data analysis

**Fig 1:**
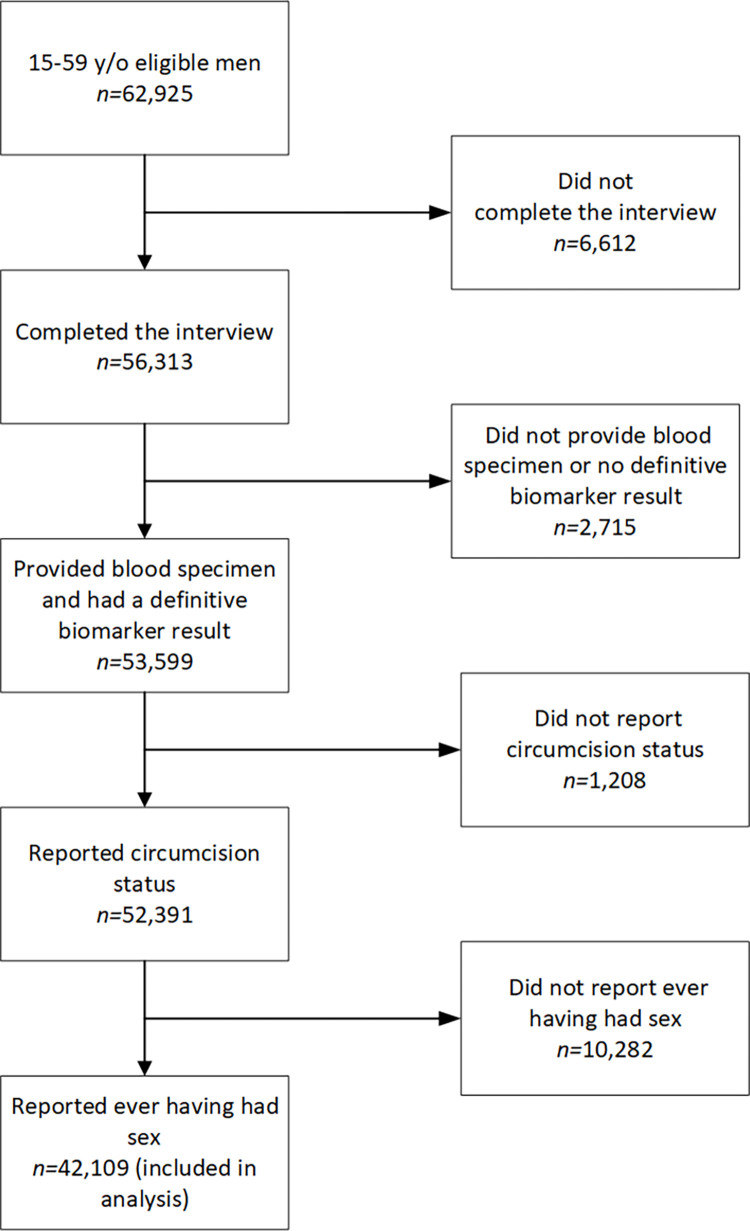
Flow chart of participants who met inclusion/exclusion criteria and the number of individuals in the analysis.

All estimates were weighted to adjust for probability of selection, nonresponse and noncoverage. Normalized weights in Rwanda were rescaled to the population weights for consistency with the other countries. For comparability across countries, only men 15–59 years of age who reported ever having had sex, their circumcision status, and participated in the biomarker portion of the survey were included (Fig 1). All other male participants were excluded from this analysis. Those who reported that their circumcision was conducted by a physician, clinical officer, midwife, or nurse were categorized as medically circumcised. Participants who reported that their circumcision was performed by a traditional practitioner/circumciser, religious leader, school personnel, family member or friend were categorized as non-medically circumcised. Circumcision type was distinguished because the amount of foreskin removed in non-medical circumcision is not uniform across settings and the protective effects of this circumcision type may be lower compared to medically circumcised men [24].

Taylor-series linearization was used to estimate variances. Summary statistics describing demographic characteristics and disease prevalence by circumcision status are presented. The main exposures of interest were circumcision status and circumcision type, medical or non-medical. The primary outcomes of interest were HIV, HBV infection (assessed with hepatitis B surface antigen (HBsAg) test), active syphilis infection, and ever syphilis infection. In Zimbabwe, Zambia, Uganda, and Tanzania, rapid and confirmatory testing was used for syphilis infection for the detection of antibodies against non-Treponemal and Treponema pallidum antigens. The test distinguished between active syphilis infection (positive for both Treponemal and non-Treponemal antibodies) and for previous infection (positive for Treponemal antibodies only).

To isolate the independent effect of the exposure variables of interest covariates were adjusted for in the multivariable models including age (in 5-year age groups), marital status, education, residence (rural or urban), country, and number of sexual partners in the past 12 months. To assess the statistical significance between circumcision status and HIV, HBV, active and ever syphilis infection, separate univariate and multivariable logistic regression models were fitted for each outcome. Odds ratios (ORs) and adjusted ORs (aORs) are presented for each association and the precision of estimates are characterized with 95% confidence intervals (CIs), with a *P* value of <0.05 considered statistically significant. Prior to finalizing the multivariable models, effect modification was assessed by including interaction terms in our regression models and adjusting for variables if the *P* values for the interaction terms were significantly different. Multi-collinearity was assessed between HIV, syphilis, and HBV infection, and variance inflation factors indicated minimal reductions on the precision of the estimated coefficients. Analysis was performed using Stata version 16 software [25].

## Results

### Characteristics of participants

From 2015 to 2019, among the 42,109 participants included in our analysis, over half, 54.1% were circumcised (Table 1). Just over a third, 34.4%, of men were medically circumcised and 19.7% were non-medically circumcised. Among all countries, Tanzania had the majority, 79.7% of males circumcised, and 50.8% were medically circumcised. Zimbabwe had the lowest proportion overall of circumcised males at 12.7% (Table 1). The 20–24-year age group had the highest proportion, 21.4%, of circumcised men among all age groups, and the 55–59-year age group had the lowest at 3.4% (Table 2). The majority, 68.0%, of uncircumcised men and 58.2%, of circumcised men were married or living with a partner. Level of education was similar by circumcision status with 54.6% of uncircumcised and 56.3% of circumcised men reporting primary school as their highest level of education completed. A higher proportion, 75.7%, of uncircumcised men were categorized as living in a rural area compared to 60.3%, of circumcised men (Table 2).

**Table 1:** Circumcision status among 15–59-year-old males in 5 Eastern and Southern African Countries, PHIA survey data, 2015–2019.

*Country*	Male circumcision status			
	Uncircumcised, n (%) [95% CI]	Total circumcised*, n (%) [95% CI]	Non-medically circumcised^†^, n (%) [95% CI]	Medically circumcised^§^, n (%) [95% CI]
**Rwanda (n = 9,760)**	5,614 (60.0)	4,146 (40.0)	267 (2.6)	3,879 (37.4)
	[57.9, 62.0]	[38.0, 42.1]	[2.2, 3.2]	[35.5, 39.3]
**Tanzania (n = 10,174)**	3,015 (20.3)	7,159 (79.7)	2,276 (29.0)	4,883 (50.8)
	[18.6, 22.1]	[77.9, 81.4]	[26.9, 31.0]	[48.6, 53.0]
**Uganda (n = 9,822)**	5,438 (55.6)	4,384 (44.4)	2,406 (22.4)	1,978 (22.0)
	[54.1, 57.2]	[42.8, 46.0]	[20.8, 24.1]	[20.8, 23.2]
**Zambia (n = 6,441)**	4,596 (72.7)	1,845 (27.3)	529 (6.5)	1,316 (20.8)
	[71.1, 74.2]	[25.8, 28.9]	[5.6, 7.5]	[19.5, 22.2]
**Zimbabwe (n = 5,912)**	5,152 (87.3)	760 (12.7)	157 (2.5)	603 (10.3)
	[86.1, 88.3]	[11.7, 13.9]	[2.0, 3.1]	[9.4, 11.3]
**Total (n = 42,109)**	**23,815 (45.9)**	**18,294 (54.1)**	**5,635 (19.7)**	**12,659 (34.4)**
	**[44.7, 47.1]**	**[52.9, 55.3]**	**[18.7, 20.7]**	**[33.3, 35.5]**

*Total circumcised includes both non-medical and medically circumcised male participants.

†Non-medically circumcised = self-reported circumcision performed by traditional practitioner/circumciser, religious leader, initiation school personnel, family member/relative, or friend.

^§^Medically Circumcised = self-reported circumcision performed by physician, clinical officer, nurse, or midwife.

Abbreviation: PHIA = Population-based HIV Impact Assessment.

All percentages presented in this table were weighted to adjust for probability of selection, nonresponse and noncoverage.

The sum of the sample sizes for a given classification may be less than the total sample size because of missing responses to the classification variable.

**Table 2:** Pooled demographic characteristics by male circumcision status, from 5 Eastern and Southern African countries*, PHIA survey data, 2015–2019.

Characteristic		Uncircumcised, n (%)	Total circumcised, n (%)	Non-medically circumcised†, n (%)	Medically circumcised§, n (%)	Total, n (%)
**Age**	15–19	2,156 (10.1)	2,319 (12.2)	573 (11.0)	1,746 (12.8)	4,475 (11.2)
	20–24	3,319 (16.1)	3,734 (21.4)	883 (17.4)	2,851 (23.6)	7,053 (18.9)
	25–29	3,354 (15.2)	3,194 (17.6)	814 (15.4)	2,380 (18.9)	6,548 (16.5)
	30–34	3,559 (14.7)	2,620 (14.1)	774 (13.8)	1,846 (14.2)	6,179 (14.4)
	35–39	3,305 (13.1)	2,112 (11.2)	668 (11.8)	1,444 (10.8)	5,417 (12.0)
	40–44	2,715 (10.9)	1,585 (8.5)	607 (9.8)	978 (7.8)	4,300 (9.6)
	45–49	2,198 (8.3)	1,182 (6.8)	562 (9.5)	620 (5.2)	3,380 (7.5)
	50–54	1,712 (6.4)	913 (4.9)	436 (6.6)	477 (3.9)	2,625 (5.6)
	55–59	1,497 (5.3)	635 (3.4)	318 (4.6)	317 (2.7)	2,132 (4.3)
	**Total**	**23,815 (100.0)**	**18,294 (100.0)**	**5,635 (100.0)**	**12,659 (100.0)**	**42,109 (100.0)**
**Marital Status**	Never married	5,579 (24.8)	6,601 (34.4)	1,344 (25.7)	5,257 (39.5)	12,180 (30.0)
	Married or living together	16,611 (68.0)	10,426 (58.2)	3,755 (65.5)	6,671 (54.0)	27,037 (62.7)
	Divorced or separated	1,365 (6.3)	1,114 (6.8)	469 (7.9)	645 (6.2)	2,479 (6.6)
	Widowed	226 (0.9)	120 (0.6)	57 (0.9)	63 (0.4)	346 (0.7)
	**Total**	**23,781 (100.0)**	**18,261 (100.0)**	**5,625 (100.0)**	**12,636 (100.0)**	**42,042 (100.0)**
**Education**	No education	1,699 (7.1)	874 (5.8)	388 (8.6)	486 (4.2)	2,573 (6.4)
	Primary	13,088 (54.6)	9,467 (56.3)	3,473 (65.6)	5,994 (51.0)	22,555 (55.5)
	Secondary	7,571 (31.5)	6,031 (28.8)	1,446 (21.3)	4,585 (33.1)	13,602 (30.0)
	Higher	1,402 (6.9)	1,902 (9.1)	323 (4.5)	1,579 (11.8)	3,304 (8.1)
	**Total**	**23,760 (100.0)**	**18,274 (100.0)**	**5,630 (100.0)**	**12,644 (100.0)**	**42,034 (100.0)**
**Primary residence**	Urban	5,242 (24.3)	7,078 (39.7)	1,682 (32.2)	5,396 (43.9)	12,320 (32.6)
	Rural	18,571 (75.7)	11,216 (60.3)	3,953 (67.8)	7,263 (56.1)	29,789 (67.4)
	**Total**	**23,815 (100.0)**	**18,294 (100.0)**	**5,635 (100.0)**	**12,659 (100.0)**	**42,109 (100.0)**
**No. of sexual partners in the past 12 months**	0–1 partner	18,716 (77.6)	13,040 (69.2)	3,779 (65.9)	9,261 (71.1)	31,756 (73.1)
	≥2 partner	4,942 (22.4)	5,123 (30.8)	1,871 (34.1)	3,306 (28.9)	10,065 (26.9)
	**Total**	**23,658 (100.0)**	**18,163 (100.0)**	**5,596 (100.0)**	**12,567 (100.0)**	**41,821 (100.0)**

*PHIA data were included from Uganda, Tanzania, Rwanda, Zambia, Zimbabwe.

^†^ Non-medically circumcised = self-reported circumcision performed by traditional practitioner/circumciser, religious leader, initiation school personnel, family member/relative, or friend.

§ Medically Circumcised = self-reported circumcision performed by physician, clinical officer, nurse, or midwife.

Abbreviation: PHIA = Population-based HIV Impact Assessment.

All percentages presented in this table were weighted to adjust for probability of selection, nonresponse, and noncoverage.

The sum of the sample sizes for a given classification may be less than the total sample size because of missing responses to the classification variable.

### Male circumcision and syphilis

#### Active syphilis infection

Of the 32,349 sexually active men tested for syphilis, 533 tested positive for active syphilis. The overall prevalence of active syphilis was 1.5% (95% CI, 1.3–1.7), 2.1% (95% CI, 1.8–2.3) among uncircumcised men, and 1.0% (95% CI, 0.8–1.3) among circumcised men (Table 3). Among circumcised men, prevalence of active syphilis ranged from 0.5% (95% CI, 0.2–1.7) in Zimbabwe to 2.7% (95% CI, 1.9–3.7) in Zambia. This was similar for uncircumcised men, with prevalence of active syphilis ranging from 0.7% (95% CI, 0.5–1.0) in Zimbabwe to 3.1% (95% CI, 2.6–3.7) in Zambia. Circumcised men had 33.0% lower odds of having an active syphilis infection compared to uncircumcised men, after controlling for covariates (95% CI, 0.52–0.87, *P* < 0.0001) (Table 4). We investigated the relationship between active syphilis infection and circumcision by type and found that non-medically circumcised men had 38.0% lower odds of having an active syphilis infection (95% CI, 0.45–0.85, *P* < 0.0001) compared to uncircumcised men, after controlling for covariates (Table 5).

**Table 3:** Weighted prevalence of HIV and STI infections by male circumcision status, from 5 Eastern and Southern African countries, PHIA survey data, 2015–2019.

	Active Syphilis Infection, n (%) [95% CI]				Ever Syphilis Infection, n (%) [95% CI]				HIV+, n (%) [95% CI]				Hepatitis B+†‡, n (%) [95% CI]			
Country	U Circ.	Total Circ.	NM Circ.	M Circ.	U Circ.	Total Circ.	NM Circ.	M Circ.	U Circ.	Total Circ.	NM Circ.	M Circ.	U Circ.	Total Circ.	NM Circ.	M Circ.
**Rwanda**	NA	NA	NA	NA	NA	NA	NA	NA	197 (3.4)	72 (1.7)	5 (1.4)	67 (1.7)	23 (2.5)	18 (3.5)	4 (12.8)	14 (2.6)
									[2.9–4.0]	[1.3–2.2]	[0.6–3.5]	[1.4–2.3]	[1.4–4.4]	[2.0–6.1]	[5.4–27.4]	[1.3–5.3]
**Tanzania**	49 (1.7)	48 (0.7)	17 (0.8)	31 (0.7)	257 (8.5)	365 (4.8)	140 (5.9)	225 (4.2)	226 (7.7)	266 (3.1)	75 (2.6)	191 (3.4)	5 (7.5)	8 (4.0)	3 (5.8)	5 (2.6)
	[1.2–2.4]	[0.5–1.0]	[0.5–1.3]	[0.4–1.1]	[7.3–9.9]	[4.2–5.5]	[4.9–7.2]	[3.5–5.1]	[6.5–9.0]	[2.6–3.6]	[1.9–3.4]	[2.8–4.0]	[2.7–19.4]	[1.6–9.7]	[1.6–18.9]	[0.8–7.9]
**Uganda**	133 (2.5)	69 (1.6)	35 (1.4)	34 (1.7)	362 (6.4)	266 (5.8)	158 (6.2)	108 (5.4)	351 (6.8)	164 (3.8)	96 (4.2)	68 (3.4)	349 (5.8)	258 (5.6)	136 (5.7)	122 (5.6)
	[2.1–3.0]	[1.2–2.0]	[1.0–2.0]	[1.2–2.5]	[5.7–7.2]	[5.0–6.7]	[5.2–7.4]	[4.4–6.7]	[6.1–7.6]	[3.2–4.4]	[3.3–5.2]	[2.7–4.3]	[5.2–6.6]	[5.0, 6.3]	[4.8–6.7]	[4.6–6.7]
**Zambia**	147 (3.1)	46 (2.7)	16 (3.6)	30 (2.4)	359 (7.5)	112 (5.9)	37 (7.4)	75 (5.4)	574 (11.9)	125 (6.9)	38 (8.2)	87 (6.5)	327 (7.2)	162 (8.6)	42 (8.5)	120 (8.6)
	[2.6–3.7]	[1.9–3.7]	[2.1–6.2]	[1.6–3.5]	[6.7–8.5]	[4.7–7.3]	[5.0–10.9]	[4.3–6.9]	[10.9–13.0]	[5.7–8.3]	[5.9–11.4]	[5.2–8.0]	[6.4–8.2]	[7.3–10.0]	[6.4–11.1]	[7.2–10.3]
**Zimbabwe**	38 (0.7) [0.5–1.0]	3 (0.5) [0.2–1.7]	2 (1.6) [0.4–7.1]	1 (0.3) [0.0–1.8]	155 (2.7)	8 (1.2)	5 (3.8)	3 (0.6)	863 (14.5)	70 (7.7)	30 (15.6)	40 (5.8)	NA	NA	NA	NA
					[2.3–3.2]	[0.6–2.6]	[1.4–9.8]	[0.2–2.1]	[13.5–15.6]	[6.0–9.8]	[11.2–21.3]	[4.1–8.2]				
**Total by circumcision status**	**367 (2.1)**	**166 (1.0)**	**70 (1.1)**	**96 (1.0)**	**1133 (6.2)**	**751 (5.0)**	**340 (6.0)**	**411 (4.4)**	**2211 (9.0)**	**697 (3.5)**	**244 (3.4)**	**453 (3.5)**	**704 (6.0)**	**446 (4.6)**	**185 (5.9)**	**261 (3.7)**
	**[1.8–2.3]**	**[0.8–1.3]**	**[0.8–1.4]**	**[0.7–1.3]**	**[5.8–6.7]**	**[4.6–5.5]**	**[5.2–6.9]**	**[3.8–5.1]**	**[8.5–9.4]**	**[3.1–3.8]**	**[2.9–4.0]**	**[3.1–4.0]**	**[4.8–7.3]**	**[2.8–7.6]**	**(2.6–13.2)**	**[2.2–6.0]**
**Overall weighted prevalence**	**533 (1.5)**				**1,884 (5.6)**				**2,908 (6.0)**				**1,150 (5.2)**			
	**[1.3–1.7]**				**[5.3–5.9]**				**[5.7–6.3]**				**[3.9–6.8]**			

^†^ Zimbabwe is not included in this estimate because it did not collect information on Hepatitis B infection among its study participants.

‡Hepatitis B+ refers to a positive hepatitis B surface antigen (HBsAg).

§Rwanda is not included in this estimate because it did not collect information on active or lifetime (ever) syphilis infections among its study participants.

Abbreviations: U Circ. = uncircumcised; Circ. = circumcision; NM Circ. = non-medically circumcised; M Circ. = medically circumcised; PHIA = Population-based HIV Impact Assessment.

*Non-medically circumcised = self-reported circumcision performed by traditional practitioner/circumciser, religious leader, initiation school personnel, family member/relative, or friend.

*Medically Circumcised = self-reported circumcision performed by physician, clinical officer, nurse, or midwife.

All percentages presented in this table were weighted to adjust for probability of selection, nonresponse, and noncoverage.

The sum of the sample sizes for a given classification may be less than the total sample size because of missing responses to the classification variable.

**Table 4:** Association between male circumcision and HIV and STI infections, from 5 Eastern and Southern African countries*, PHIA survey data, 2015–2019.

*HIV and STI infection*		Active Syphilis^§^				Ever Syphilis^§^				HIV+				Hepatitis B+ǂ‡			
		Crude		Adjusted†		Crude		Adjusted†		Crude		Adjusted†		Crude		Adjusted†	
		OR (95% CI)	*P* value	OR (95% CI)	*P* value	OR (95% CI)	*P* value	OR (95% CI)	*P* value	OR (95% CI)	*P* value	OR (95% CI)	*P* value	OR (95% CI)	*P* value	OR (95% CI)	*P* value
Circumcision status	Circumcised	**0.49 (0.39–0.62)**	**0.00**	**0.67 (0.52–0.87)**	**0.00**	**0.80 (0.70–0.91)**	**0.00**	**0.85 (0.73–0.98)**	**0.03**	**0.36 (0.33–0.41)**	**0.00**	**0.53 (0.47–0.61)**	**0.00**	0.76 (0.42–1.39)	0.37	0.91 (0.52–1.60)	0.75
	Uncircumcised	Ref	-	-	-	-	-	-	-	-	-	-	-	-	-	-	-

* PHIA data were included from Uganda, Tanzania, Rwanda, Zambia, Zimbabwe.

†Adjusted for age, marital status, education, residence, country, and number of sexual partners in the past 12 months.

ǂ Zimbabwe did not collect information on Hepatitis B infection among participants.

‡Hepatitis B+ refers to a positive hepatitis B surface antigen (HBsAg).

^§^ Rwanda did not collect information on active or lifetime (ever) syphilis infections among participants.

Abbreviation: PHIA = Population-based HIV Impact Assessment.

**Table 5:** Association between male circumcision and HIV and STI infections, from 5 Eastern and Southern African countries*, PHIA survey data, 2015–2019.

*HIV and STI infection*		Active Syphilis^§^				Ever Syphilis^§^				HIV+				Hepatitis B+^¶^‡			
		Crude		Adjusted†		Crude		Adjusted†		Crude		Adjusted†		Crude		Adjusted†	
		OR (95% CI)	*P* value	OR (95% CI)	*P* value	OR (95% CI)	*P* value	OR (95% CI)	*P* value	OR (95% CI)	*P* value	OR (95% CI)	*P* value	OR (95% CI)	*P* value	OR (95% CI)	*P* value
Circumcision status	Medically circumcised**	**0.47 (0.34–0.64)**	**0.00**	0.72 (0.52–1.01)	0.06	**0.70 (0.59–0.82)**	**0.00**	0.84 (0.69–1.02)	0.07	**0.37 (0.32–0.42)**	**0.00**	**0.61 (0.53–0.71)**	**0.00**	0.60 (0.33–1.07)	0.08	0.70 (0.36–1.36)	0.29
	Non-medically circumcisedǂ	**0.53 (0.39–0.71)**	**0.00**	**0.62 (0.45–0.85)**	**0.00**	0.96 (0.82–1.13)	0.65	0.86 (0.72–1.01)	0.07	**0.36 (0.30–0.43)**	**0.00**	**0.44 (0.36–0.53)**	**0.00**	0.99 (0.39–2.5)	0.99	1.18 (0.50–2.81)	0.71
	Uncircumcised	Ref	-	-	-	-	-	-	-	-	-	-	-	-	-	-	-

*PHIA data were included from Uganda, Tanzania, Rwanda, Zambia, Zimbabwe.

^¶^ Zimbabwe is not included in this estimate because it did not collect information on Hepatitis B infection among its study participants.

‡ Hepatitis B+ refers to a positive hepatitis B surface antigen (HBsAg).

†Adjusted for age, marital status, education, residence, country, and number of sexual partners in the past 12 months.

^§^ Rwanda is not included in this estimate because it did not collect information on active or lifetime (ever) syphilis infections among its study participants.

** Medically circumcised was defined as men who reported that their circumcision was conducted by a physician, clinical officer, midwife, or nurse.

ǂ Non-medically circumcised was defined as men who reported that their circumcision was performed by a traditional practitioner/circumciser, religious leader, initiation school personnel, family member or friend.

Abbreviation: PHIA = Population-Based HIV Impact Assessment.

#### Ever syphilis infection

Among the 32,349 sexually active men who tested for syphilis, 1,884 tested positive for ever having had a syphilis infection, resulting in a prevalence of 5.6% (95% CI, 5.3–5.9) (Table 3). Prevalence of ever having had syphilis was 6.2% (95% CI, 5.8–6.7) among uncircumcised men and 5.0% (95% CI, 4.6–5.5) among circumcised men. Among circumcised men, prevalence of ever having had syphilis ranged from 1.2% (95% CI, 0.6–2.6) in Zimbabwe to 5.9% (95% CI, 4.7–7.3) in Zambia. Similarly, among uncircumcised men, prevalence of ever having had a syphilis infection ranged from 2.7% (95% CI, 2.3–3.2) in Zimbabwe to 8.5% (95% CI, 7.3–9.9) in Tanzania. After controlling for covariates, circumcised men had 15.0% lower odds of ever having had a syphilis infection compared to uncircumcised men (95% CI, 0.73–0.98, *P* = 0.03). When we looked at circumcision by type, men who were medically circumcised had 30.0% lower odds of ever having had a syphilis infection (95% CI, 0.59–0.82, *P* < 0.0001); however this effect did not remain significant in the adjusted model (aOR, 0.84, 95% CI, 0.69–1.02, *P* = 0.07) (Table 5). Non-medical circumcision was not associated with ever having had a syphilis infection when compared to uncircumcised men, in either the crude or adjusted model.

#### Male circumcision and HIV

Of the 42,109 sexually active men tested for HIV, 2,908 tested positive, resulting in a prevalence of 6.0% (95% CI, 5.7–6.3) (Table 3). Prevalence of HIV was 9.0% (95% CI, 8.5–9.4) among uncircumcised men and 3.5% (95% CI, 3.1–3.8) among circumcised men. Among circumcised men, prevalence of HIV ranged from 1.7% (95% CI, 1.3–2.2) in Rwanda to 7.7% (95% CI, 6.0–9.8) in Zimbabwe. Similarly, among uncircumcised men, prevalence of HIV ranged from 3.4% (95% CI, 2.9–4.0) in Rwanda to 14.5% (95% CI, 13.5–15.6) in Zimbabwe. Circumcised men had 48.0% lower odds of testing positive for HIV compared to uncircumcised men in the adjusted model (95% CI, 0.47–0.61, *P* < 0.0001) (Table 4). When we examined circumcision by type, both medical and non-medical circumcision were associated with a reduction in odds in testing positive for HIV infection compared to uncircumcised men (aOR, 0.61, 95% CI, 0.53–0.71, *P* < 0.0001; aOR, 0.44, 95% 0.36–0.53, *P* < 0.0001) respectively (Table 5).

#### Male circumcision and HBV infection

Of the 17,807 sexually active men tested for HBsAg in four countries, 1,150 tested positive, resulting in a prevalence of 5.2% (95% CI, 3.9–6.8) (Table 3). Prevalence of HBsAg was 6.0% (95% CI, 4.8–7.3) among uncircumcised men and 4.6% (95% CI, 2.8–7.6) among circumcised men. Among circumcised men, prevalence of HBsAg ranged from 3.5% (95% CI, 2.0–6.1) in Rwanda, to 8.6% (95% CI, 7.3–10.0) in Zambia. Non-medically circumcised men in Rwanda had the highest prevalence of HBsAg among any country at 12.8% (95% CI, 5.4–27.4). Among uncircumcised men, prevalence of HBsAg ranged from 2.5% in Rwanda to 7.5% in Tanzania. In both the crude and adjusted models, the association between circumcision and testing positive for HBsAg was not significant (*P* > 0.05) (Table 4). After adjusting for covariates, circumcised men had 9.0% lower odds of testing positive for HBsAg compared to uncircumcised men, but this association was not significant (95% CI, 0.52–1.60; *P* = 0.75) (Table 4). When we looked at circumcision by type, medical or non-medical, neither were significantly associated with HBsAg infection when compared to uncircumcised men (Table 5). Non-medically circumcised men had 1.1% higher odds of testing positive for HBsAg compared to uncircumcised men, but this was not significant (95% CI, 0.50–2.81, *P* = 0.71).

## Discussion

In our analysis, multivariable models demonstrated that male circumcision significantly reduced the odds of testing positive for a syphilis infection or HIV. These findings, along with previous research, suggest that syphilis and HIV are significantly lower among circumcised men compared to uncircumcised men [8–13,35,36]. Given the cross-sectional study design of the PHIA surveys, our analysis could not distinguish if syphilis infection predated circumcision. Globally, syphilis infection has predominantly been acquired among men who have sex with men (MSM), however, the PHIA surveys did not measure MSM behavior and therefore we cannot make any inferences about syphilis infection and male circumcision among MSM [26,27].

Non-medical circumcision status was independently associated with a protective effect against active syphilis and HIV infection. While our finding that non-medical circumcision may be protective against HIV and syphilis is encouraging, the findings should be interpreted with caution. There is potential for misclassification of self-reported circumcision method given that assessment of circumcision did not include an external examination of whether male participant’s circumcision was partial or complete. The amount of foreskin removed in non-medical circumcision is not uniform across settings and the effect of medical circumcision could have potentially been stronger if circumcision status had been verified. The benefits of non-medical circumcision are inconsistent with previous epidemiological studies demonstrating that the protective effects of non-medical circumcision for HIV and STI prevention vary [28,34]. Additionally, evidence demonstrates a higher risk of adverse events from non-medical circumcision compared to medical circumcision [29].

Our analysis found that uncircumcised men had a slightly higher prevalence of HBV infection at 6.0%, compared to circumcised men at 4.6%. However, there was no significant difference in HBsAg positivity between circumcised and uncircumcised men. In countries with high hepatitis B endemicity, perinatal and parenteral horizontal transmission through exposure to infected blood are the most common risk factors for HBV infection [30]. Perinatal transmission is considered the greatest contributing factor to high prevalence of chronic HBV infection as 90% of children under 1 year of age infected with HBV progress to chronic HBV infection compared to less than 10% of adults [31,32]. Previous analysis has demonstrated that chronic HBV infection is lower in men who are medically circumcised (OR 0.53, 95% CI, 0.3–0.9) [33]. Epidemiological studies have also indicated that a history of circumcision in some settings may be a risk factor for HBV, likely due to non-medical circumcisions performed in unhygienic settings [34,35]. However, most studies do not distinguish type of circumcision. Our analysis found that non-medically circumcised men at a higher prevalence of HBV infection at 5.9% compared to medically circumcised men at 3.7%. Additional research that tests men for hepatitis B core antigen (anti-HBc) and HBsAg before and after circumcision and distinguishes circumcision type is needed to better understand the association between circumcision and HBV. Findings from this analysis can inform VMMC programs in other countries in Eastern and Southern Africa, particularly those that have been prioritized for VMMC.

### Limitations

There are limitations to this analysis that should be taken into consideration. First, the PHIA surveys use a cross-sectional design which limits the ability to explain the cause-and-effect relationship between circumcision and STIs. Second, circumcision status was self-reported and may be subject to misclassification. The accuracy of reported method of circumcision, medical vs. non-medical, is also limited to participants’ interpretation of the question, response bias, and recollection of who performed their circumcision [36–38]. Furthermore, given that less than 10% of adults with HBV progress to chronic HBV infection, our analysis did not assess whether circumcision protected against HBV infection by testing for anti-HBc, a marker of previous exposure to HBV. This may potentially explain the non-significant association between circumcision by type and HBV and syphilis infection. Additionally, the variation in years during which the five PHIA surveys took place, ranging from 2015–2019, raises the possibility of temporal differences in male circumcision coverage and the implementation of other HIV prevention interventions. Differences in the effect of male circumcision on STI infection by country may also be impacted by unknown differences between countries. Finally, the findings from this analysis were limited to 5 countries and the result may not be generalizable to other populations where risk of HIV and STIs differ.

Findings from this analysis suggest that male circumcision significantly reduces prevalence of syphilis infection, further supporting that VMMC serves a broader public health function in STI prevention and control beyond the primary goal of HIV prevention. As countries prioritized for VMMC progress towards sustainability, findings from this analysis are useful for advocating for the benefits of VMMC not only lowering risk of HIV infection, but also protecting against other STIs.
